# Dysregulation of Microtubule Stability Impairs Morphofunctional Connectivity in Primary Neuronal Networks

**DOI:** 10.3389/fncel.2017.00173

**Published:** 2017-06-22

**Authors:** Peter Verstraelen, Jan R. Detrez, Marlies Verschuuren, Jacobine Kuijlaars, Rony Nuydens, Jean-Pierre Timmermans, Winnok H. De Vos

**Affiliations:** ^1^Laboratory of Cell Biology and Histology, Department of Veterinary Sciences, University of AntwerpAntwerp, Belgium; ^2^Biomedical Research Institute, University of HasseltDiepenbeek, Belgium; ^3^Janssen Research and Development, Division of Janssen Pharmaceutica N.V.Beerse, Belgium; ^4^Department of Molecular Biotechnology, University of GhentGhent, Belgium

**Keywords:** microtubule, primary hippocampal neuron, neuronal network, synapse, P301L, Tau aggregation, high-content microscopy, live cell imaging

## Abstract

Functionally related neurons assemble into connected networks that process and transmit electrochemical information. To do this in a coordinated manner, the number and strength of synaptic connections is tightly regulated. Synapse function relies on the microtubule (MT) cytoskeleton, the dynamics of which are in turn controlled by a plethora of MT-associated proteins, including the MT-stabilizing protein Tau. Although mutations in the Tau-encoding *MAPT* gene underlie a set of neurodegenerative disorders, termed tauopathies, the exact contribution of MT dynamics and the perturbation thereof to neuronal network connectivity has not yet been scrutinized. Therefore, we investigated the impact of targeted perturbations of MT stability on morphological (e.g., neurite- and synapse density) and functional (e.g., synchronous calcium bursting) correlates of connectivity in networks of primary hippocampal neurons. We found that treatment with MT-stabilizing or -destabilizing compounds impaired morphofunctional connectivity in a reversible manner. We also discovered that overexpression of *MAPT* induced significant connectivity defects, which were accompanied by alterations in MT dynamics and increased resistance to pharmacological MT depolymerization. Overexpression of a *MAPT* variant harboring the P301L point mutation in the MT-binding domain did far less, directly linking neuronal connectivity with Tau's MT binding affinity. Our results show that MT stability is a vulnerable node in tauopathies and that its precise pharmacological tuning may positively affect neuronal network connectivity. However, a critical balance in MT turnover causes it to be a difficult therapeutic target with a narrow operating window.

## Introduction

Hippocampal neurons exhibit an extraordinary morphology with long axons and complex dendritic trees to exert their function as information integrators and transmitters. Arguably, the most important cytoskeletal components that support this architecture are the microtubules (MTs). MTs are polarized multimers of α- and β-tubulin heterodimers which are not rigid but show phases of growing and shrinking, a process known as dynamic instability. While the bulk of neuronal MTs is more stable (average growth at plus end v^+^ = 0.2 μm/s) than MTs of dividing cells (v^+^ = 0.5 μm/s; Stepanova et al., 2003), there is also an important fraction of shorter, unstable MTs. Even within the same MT, domains can be discriminated that show differential stability, composition and interaction with MT-associated protein (Baas et al., 2016). MT plus-end tracking proteins (+TIPs) interact with numerous cytoplasmic signaling proteins to regulate MT dynamics (Dent and Baas, 2014). Other mechanisms of regulation include tubulin post-translational modifications, binding and bundling of individual MTs by MT-associated proteins, and the intrinsic polarity of MT arrays. The latter is very different in axons than it is in dendrites (Wloga and Gaertig, 2011; Kapitein and Hoogenraad, 2015).

Motor-dependent trafficking of mRNA, (antero- and retrograde signaling) proteins, synaptic vesicle precursors and organelles along MTs is crucial for synapse formation, elimination and plasticity (van den Berg and Hoogenraad, 2012). While stable MTs provide structural support, and facilitate cargo transport along the neurites, a subset of unstable MTs is directly involved in local signaling at the synapse (Gardiner et al., 2011; Dent, 2017). More specifically, dynamic MTs physically enter dendritic spines in response to synaptic NMDA receptor activity and modulate spine morphology via interaction with F-actin (Jaworski et al., 2009; Merriam et al., 2013). Furthermore, proteins such as Ca^2+^/calmodulin-dependent protein kinase II and Tau can translocate between dendritic MTs and adjacent spines in response to synaptic activity (Lemieux et al., 2012; Frandemiche et al., 2014; McVicker et al., 2015).

Given their prominent role in synapse function, it is not surprising that dysregulation of neuronal MT dynamics is associated with mental and cognitive symptoms (Zempel and Mandelkow, 2015; Marchisella et al., 2016). Many drugs used in anti-cancer treatment exert their cytostatic effect via hyper- (e.g., paclitaxel) or destabilization (e.g., nocodazole) of MTs during mitotic spindle formation. Cancer patients receiving paclitaxel not only suffer from peripheral neuropathy as a side effect, but also experience cognitive problems that may persist after cessation of the therapy (Wefel et al., 2010; Jaggi and Singh, 2012; Gornstein and Schwarz, 2014). Furthermore, it is also known that MT dysregulation contributes to the reduced dendritic complexity and synaptic density in central neurons of patients suffering from mood disorders and schizophrenia (Andrieux et al., 2006; Marchisella et al., 2016). But, perhaps the best characterized link between MT dysregulation and impaired neuronal connectivity can be found in so-called tauopathies. Tau is one of the MT-stabilizing proteins (Weingarten et al., 1975; Cleveland et al., 1977) with a role in axon elongation (Sayas et al., 2015), release of cargo from motor proteins near the synapse (Medina et al., 2016), and long-term depression of synaptic transmission (Regan et al., 2015). In tauopathies such as frontotemporal dementia and Alzheimer's disease (AD), mutations are frequently found in *MAPT*, the gene encoding Tau. These mutations are often associated with increased levels of Tau phosphorylation and decreased MT affinity (Hong et al., 1998; Dayanandan et al., 1999; Barghorn et al., 2000; von Bergen et al., 2001).

Alterations in MT stability have been reported in several animal models for CNS disorders and pharmacological MT stabilization was found to alleviate behavioral symptoms in some of these models (Andrieux et al., 2006; Barten et al., 2012; Zhang et al., 2012; Vaisburd et al., 2015). However, the relationship between MT stability and synaptic connectivity has not yet been scrutinized at the cellular level. We previously showed that primary hippocampal cultures form spontaneously active, synaptically connected neuronal networks and hence represent a valid *in vitro* model for studying morphofunctional features of neuronal connectivity (Cornelissen et al., 2013; Verstraelen et al., 2014; Detrez et al., 2016). Here, we exploit this model to gain further insight into the role of MT dynamics in neuronal connectivity using targeted pharmacological and genetic perturbations.

## Materials and methods

### Preparation of primary hippocampal cultures

This study was carried out in accordance with the recommendations of the ethical committee for animal experimentation of the University of Antwerp (approved ethical files 2013-46 and 2015-54).

**Figure 1:**
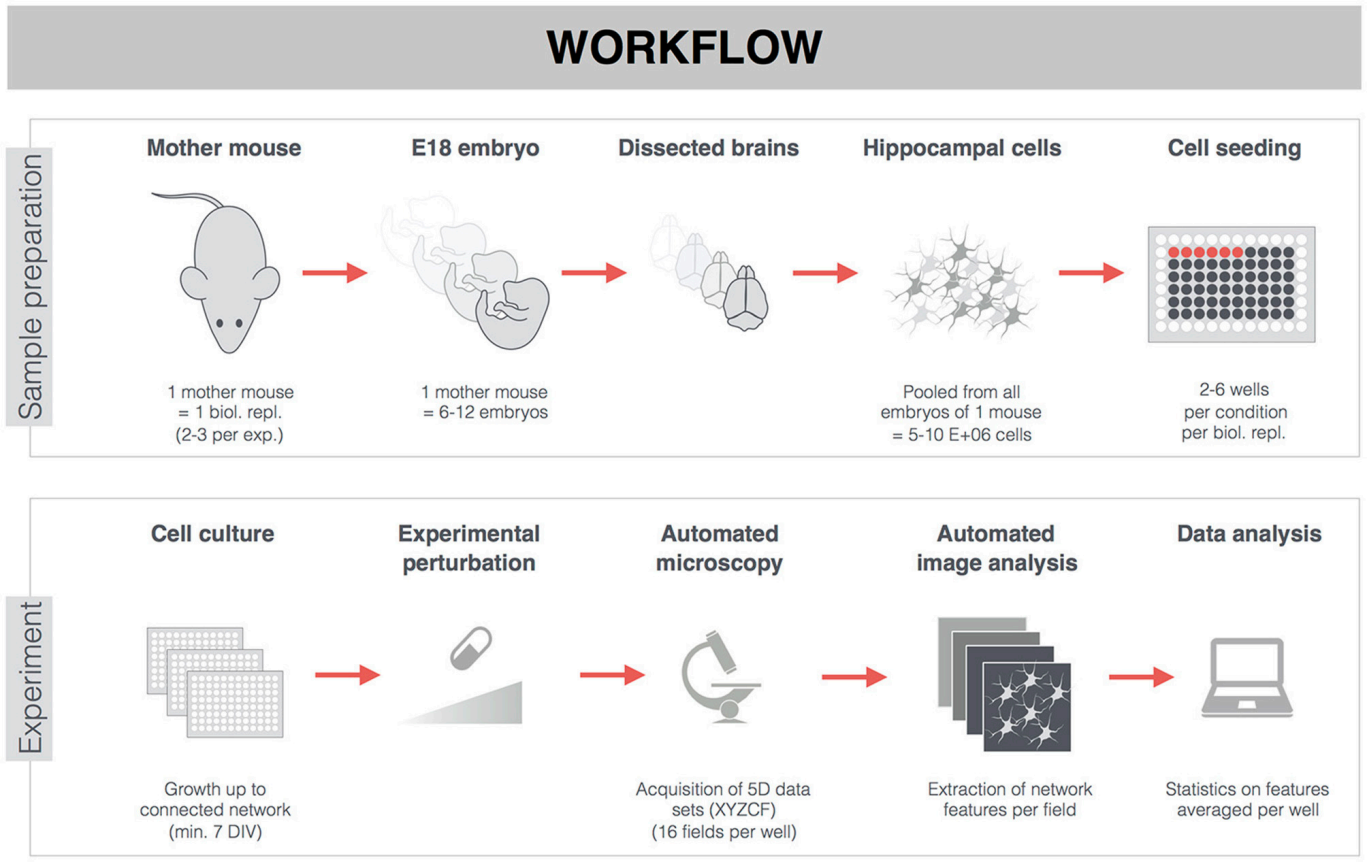
General workflow of experiments on primary hippocampal neurons. Hippocampi of E18 embryos from the same mother mouse were pooled, dissociated, and seeded in 96-well plates. Sacrificing one mother mouse with on average 9 embryos yielded 300–350 wells (15,000 cells/well). The week-separated dissection of one mother mouse was considered a biological replicate. Per treatment condition, there were 2–6 wells from 2–3 biological replicates (see also Supplemental Table 1). Though treatments (pharmacological or *MAPT* overexpression) were often started earlier, analyses were carried out on mature neuronal networks of 7-22 DIV. After fluorescent labeling, 3D (XYZ) images were automatically acquired at different positions (fields, F), and in different channels (C), thus yielding 5D datasets per well (XYZCF). Multiple features were extracted and averaged per well (e.g., when 16 fields were acquired per well) before proceeding to statistical analysis.

Hippocampi were dissected from WT E18 C57Bl6 mouse embryos in Hepes(7 mM)-buffered Hanks Balanced Salt Solution, followed by trypsin digestion (0.05%; 10 min; 37°C) and mechanical dissociation by trituration through 2 fire-polished glass pipettes with decreasing diameter. After centrifugation (5 min at 200 g), the cell pellet was resuspended in Minimal Essential Medium supplemented with 10% heat-inactivated normal horse serum and 30 mM glucose. Cells were plated in Poly-D-Lysin-coated 96-well plates (Greiner Cell coat, μClear), at 45,000 cells/cm^2^, and kept in a humidified CO_2_ incubator (37°C; 5% CO_2_). After 4 h, the medium was replaced with B27 supplemented Neurobasal medium, containing Sodium Pyruvate (1 mM), Glutamax (2 mM) and glucose (30 mM). To suppress proliferation of non-neuronal cells, arabinosylcytosine was added in 50 μl Neurobasal-B27 medium at the third day after plating. The cultures were grown without any further medium replacement until the time of analysis, with a minimum of 7 days *in vitro* (DIV) to ascertain a sufficiently connected network (Figure 1). Cell culture supplies were purchased from ThermoFisher.

### Pharmacology

Drugs were purchased from Sigma-Aldrich or obtained via an in-house J&J compound library. Paclitaxel or nocodazole (0.1–100 nM) was added at the fourth day *in vitro* (DIV). Effects were evaluated at 7 DIV without medium replacement. For acute nocodazole treatment, 1 μM was added at 7 DIV and effects were assessed after 2, 4, or 8 h. For rescue experiments, nocodazole (1 μM) was added, followed after 2 h by paclitaxel (100 nM) or epothilone D (100 nM). Fixation and calcium imaging were carried out 4 h after initial nocodazole treatment. As such, the cells were exposed to nocodazole alone for 2 h, followed by a 2 h period of the nocodazole/MT stabilizer combination. As control, DMSO was added two times, upon nocodazole as well as MT stabilizer treatment. For evaluation of the resistance to nocodazole-induced depolymerization, cultures were pre-treated with 10 nM paclitaxel or transduced with *MAPT(-P301L)-eGFP* at 3 DIV. At 10 DIV, cultures were treated with 1 μM nocodazole for 4 h and then fixed for immunocytochemistry.

### Viral transduction and treatment with K18 fibrils

AAV6 particles for the neuronal-specific (hSyn1 promoter) overexpression of human *4R-MAPT-eGFP, 4R-MAPT(P301L)-eGFP*, and *eGFP* (as AAV control) were produced as described before (Taschenberger et al., 2013; Calafate et al., 2015) and were administered at 3 DIV at MOI 100, unless indicated otherwise. Both the *MAPT* and *MAPT-P301L* overexpression constructs used in this study coded for 4-repeat Tau, which is the form that has the highest affinity for MTs (Dayanandan et al., 1999). To produce K18 fibrils, monomeric Tau K18-P301L (40 μM) was incubated for 48–72 h at 37°C in the presence of the polyanion heparin (40 μM), DTT (2 mM) and sodium acetate buffer (100 mM; pH 7.0). The solution was centrifuged (100,000 g, 1 h, 4°C) and the pellet was resuspended in sodium acetate buffer and sonicated before use. At 6 DIV, Tau aggregation was induced by adding the K18 fibrils (25 nM; Guo and Lee, 2013) to the culture medium.

### Staining and immunocytochemistry

For cytotoxicity measurement, live 7 DIV cultures were incubated with 2.5 μg/ml propidium iodide (PI, Sigma-Aldrich) and 10 μg/ml of the membrane-permeable Hoechst 33342 (Sigma-Aldrich) for 30 min at 37°C and 5% CO_2_, without removal of the culture medium. For immunocytochemistry, paraformaldehyde-fixed cultures (2%, 20 min, RT) were permeabilized with 1% Triton X-100 in blocking buffer (0.1% bovine serum albumin and 10% normal horse serum in PBS) for 10 min, followed by a 4-h incubation with the primary antibodies (Table 1) at RT in blocking buffer. After washing with PBS, secondary antibodies (Table 1) were added for 2 h. Finally, DAPI was applied to the cultures for 10 min at a concentration of 2.5 μg/ml, followed by a PBS wash. To label fibrillar Tau, cultures were stained with pentameric formyl thiophene acetic acid (pFTAA; 1 μM, 2 h) (Aslund et al., 2009; Brelstaff et al., 2015).

**Table 1:** Primary and secondary antibodies for immunocytochemistry.

**Primary antibodies**				
β-III-tubulin	Rabbit Polyclonal	Covance	PRB-435P	1/2,000
MAP2	Chicken Polyclonal	abcam	ab5392	1/5,000
Synaptophysin-I	Guinea Pig Polyclonal	Synaptic Systems	101 004	1/1,000
Acetylated α-tubulin	Mouse monoclonal	Sigma-Aldrich	T6793	1/2,000
Total Tau	Rabbit Polyclonal	Dako	A0024	1/2,000
AT8 (hyperphosphorylated Tau)	Mouse Monoclonal	ThermoFisher	MN1020	1/5,000
**Secondary antibodies**				
Donkey-anti-Mouse	FITC/Cy5	Jackson	715-095-150	1/1,000
			715-175-150	
Goat-anti-Rabbit	FITC/Cy3	Jackson	111-095-047	1/4,000
F_ab_ fragments			111-165-047	
Donkey-anti-Guinea Pig	Cy3	Jackson	706-165-148	1/1,000

### Microscopy

For cytotoxicity measurements, images were acquired on a BD pathway 435 Bioimager (20X, NA 0.75, Becton Dickinson). To minimize imaging time, these images were acquired in non-confocal mode (1 Z-plane). All images on fixed cultures were acquired in confocal mode with a spinning disk confocal microscope (40X, NA 0.95, Ultra*VIEW* VoX, PerkinElmer) or in high-throughput mode on an Opera Phenix High Content Screening System (40XW, NA 1.2, PerkinElmer). Per well, 16 frames (4 × 4) were acquired with an inter-frame gap of 500 μm. Per frame, up to 4-channel images (405, 488, 561, and 640 nm excitation) were acquired in at least 6 axial positions separated by a 1 μm spacing, thus yielding 5-dimensional image data sets (XYZCF; Figure 1). Different fluorescence channels were separated using standard excitation/emission filters and dichroic mirrors. In case of confocal imaging, maximum intensity projections were used for downstream image analysis.

### High-content image analysis

High-content image analysis scripts for immunostained neuronal networks were developed for FIJI image analysis freeware (Schindelin et al., 2012). For all workflows, images were pre-processed using a rolling ball background subtraction to correct for illumination heterogeneity. To analyze cytotoxicity, images of Hoechst 33342 counterstained cells were first smoothed by Gaussian blurring (radius 1.75 μm) and segmented using a fixed threshold. Neighboring nuclei were separated by watershed segmentation and debris was removed by size filtering of the segmented objects (minimum size of 90 μm^2^). This resulted in a set of regions of interest (ROIs) corresponding to the total number of nuclei. Dead cells were then identified as ROIs for which the average intensity in the PI channel exceeded a fixed intensity threshold. To measure neurite density after β-III-tubulin or MAP2 immunolabeling, a multi-tier approach was employed, based on MorphoNeuroNet (Pani et al., 2014; Detrez et al., 2016) (the updated script, Neuronmetrics.ijm, is available upon request). In brief, the high intensity parts of the image were extracted by an automated thresholding procedure (Isodata algorithm) yielding a first mask. To also include the finer, low-intensity parts of the cytoskeletal network, a second mask was established by local contrast enhancement (block size 1.8 μm, slope 3) followed by Laplacian edge enhancement. Both masks were combined into a single network ROI of which the surface was measured. To quantify tubulin acetylation, the intensity ratio of acetylated α- over β-III-tubulin was measured inside the network ROI. The intensity ratio of AT8 over total Tau was measured in a similar way. To assess the integrity of the MT network, the colocalization between β-III- and acetylated α-tubulin was quantified in terms of the Pearson correlation coefficient (Manders et al., 1993). Fibrillar Tau structures were identified in neurons after enhancing tube-like structures in the pFTAA channel (Tubeness plugin, sigma 3), automated thresholding (Triangle algorithm) and particle analysis (minimum size 15 μm^2^ and circularity 0.00-0.30). The fibrillary Tau load was expressed as total area of the segmented particles within the MT mask. To quantify synaptophysin spots, images were pre-processed by means of Laplace filtering (smoothing scale 0.35 μm), followed by automated thresholding (Triangle algorithm), particle size filtering (minimum size of 0.75 μm^2^) and spot counting (based on a pipeline described before; De Vos et al., 2010). Synapse density was expressed as the number of synaptophysin-I puncta per μm^2^ neurite area.

### Live cell calcium imaging

For pharmacology experiments, neurons were loaded with 2 μM Fluo-4-AM (ThermoFisher) in Neurobasal B27 medium at 37°C and 5% CO_2_. After 30 min, the medium was replaced with recording medium, containing (in mM): CaCl_2_ 0.9, MgCl_2_ 0.5, KCl 2.67, NaCl 138, KH_2_PO_4_ 1.47, Na_2_HPO_4_-7H_2_O 8, and C_6_H_12_O_6_ 10. Because MTs are known to recover from nocodazole administration upon medium exchange (Baas and Ahmad, 1992; Jaworski et al., 2009), nocodazole was also added to the recording medium when appropriate. Cells were imaged on an inverted dual spinning disk confocal microscope (UltraVIEW ERS, PerkinElmer) for 260 s, with a 25X objective lens (NA 0.80) at 2 frames per second. To distinguish neurons from non-neuronal cells, 30 μM glutamate was added during the last 20 s of calcium recording (Pickering et al., 2008). Immediately after the calcium recording, DAPI was added to allow accurate cell segmentation. For *MAPT-eGFP* overexpression experiments, a red genetically encoded calcium indicator (RGECO; Zhao et al., 2011) was introduced at 0 DIV via AAV-mediated expression under the synapsin promoter. Imaging was performed on a spinning disk confocal microscope (UltraVIEW VoX, PerkinElmer, UK) at 37°C and 5% CO_2_ and the same cultures were imaged on different DIV.

Calcium recordings were analyzed using a home-written MATLAB script (Cornelissen et al., 2013). In case of Fluo-4 imaging, all cells were segmented based on a nucleus image, after which traces of the fluorescence intensity over time were generated. Traces of non-neuronal cells were discarded based on their response to glutamate. In case of RGECO, the expression of which was limited to neurons (hSyn1 promoter), ROIs were manually drawn over neuronal cell bodies. Subsequent signal analysis returned parameters such as percentage of neurons that show at least one peak during the recording, frequency of synchronous calcium bursts and burst correlation, which is the average of the Pearson's correlation matrix between all neuron pairs in the field of view. The original script was also adapted to allow for simultaneous analysis of neuronal subpopulations (Tau aggregate-positive vs. -negative) within the same field of view.

### EB3 tracking

Cultures were transduced with AAV6 particles for the overexpression of human *4R-MAPT-eGFP, 4R-MAPT(P301L)-eGFP*, and *eGFP* (as AAV control) or treated with 10 nM paclitaxel at 3 DIV. Lentiviral particles encoding an EB3-RFP fusion protein were added at 5 DIV (LentiBrite EB3-RFP Lentiviral Biosensor, Merck Millipore, MOI 25). This transfection protocol resulted in relatively sparse (~25%) labeling of neurons and sufficiently isolated axons within the dense neuronal network. At 10 DIV, 100 nM paclitaxel (4 h) was added to some of the wells and imaging was performed on a spinning disk confocal microscope (UltraVIEW VoX, PerkinElmer) at 37°C and 5% CO_2_, 60X water immersion objective (NA 1.20). One frame was recorded every 2 s for 1 min. Image analysis was carried out in FIJI image analysis freeware (Schindelin et al., 2012), blinded for treatment conditions. For each well, at least 7 neurite segments (each >20 μm) were manually selected and kymographs were generated using the multi-kymograph plugin (line width 3). The velocity of the EB3 comets was calculated using the “read velocity from tsp” macro (http://www.embl.de/eamnet/downloads/macros/tsp050706.txt).

### Statistics

The typical workflow of the experiments is shown in Figure 1 and the number of biological (n_b_) and well (n_w_) replicates of each experiment is mentioned in the figure captions and summarized in Supplemental Table 1. One biological replicate refers to the dissection of one mother mouse (2–3 biological replicates per experiment). Biological replicates were separated in time, meaning that the breeding and housing conditions of the mice, as well as the cultivation conditions (e.g., media and well plate batches) were slightly different between biological replicates. Hippocampi from different E18 embryos from the same mother mouse were pooled after dissection (6–12 embryos per mother mouse). As such, sacrificing one mother mouse with an average of 9 embryos yielded in between 5 and 10 E06 cells. This was sufficient to seed cells in 300–350 96-well plate wells (2–6 per biological replicate per plate per treatment). When multiple measurements were done per well (e.g., 16 images were acquired per well in automated microscopy), the data were first averaged per well before executing statistical analysis.

Statistical analyses were carried out in SAS JMP Pro 12 software. Shapiro-Wilk W tests were used to check for normality (Supplemental Table 1). Since the majority of the data was not normally distributed, non-parametric tests were performed throughout the paper. Kruskal-Wallis (rank sums) tests were performed to assess the overall effect across treatments (Supplemental Table 1). For longitudinal experiments, this test was performed within each DIV and for studying the resistance to nocodazole-induced depolymerization, tests were performed within the DMSO and nocodazole group, separately. Conditional to the overall Kruskal-Wallis test, *post-hoc* tests were performed (^*^p < 0.05 and ^**^p < 0.005; Supplemental Table 1). A Steel test for comparison with control was used as the non-parametric alternative for a Dunnett test (Steel, 1959). For rescue of nocodazole-induced defects, where all pairwise comparisons were of interest, Dunn all pairs tests for joint ranks were used as the non-parametric alternative for Bonferroni tests (Dunn, 1964). The data are represented as bar charts (mean + standard deviation). No normalization was applied to suppress the variability between biological replicates, nor did we use statistical techniques such as Mixed models that accommodate for this variation. This conservative statistical testing allowed drawing robust conclusions while keeping the number of sacrificed animals to a minimum.

## Results

### Pharmacological modulation of MT stability impairs neuronal network connectivity

**Figure 2:**
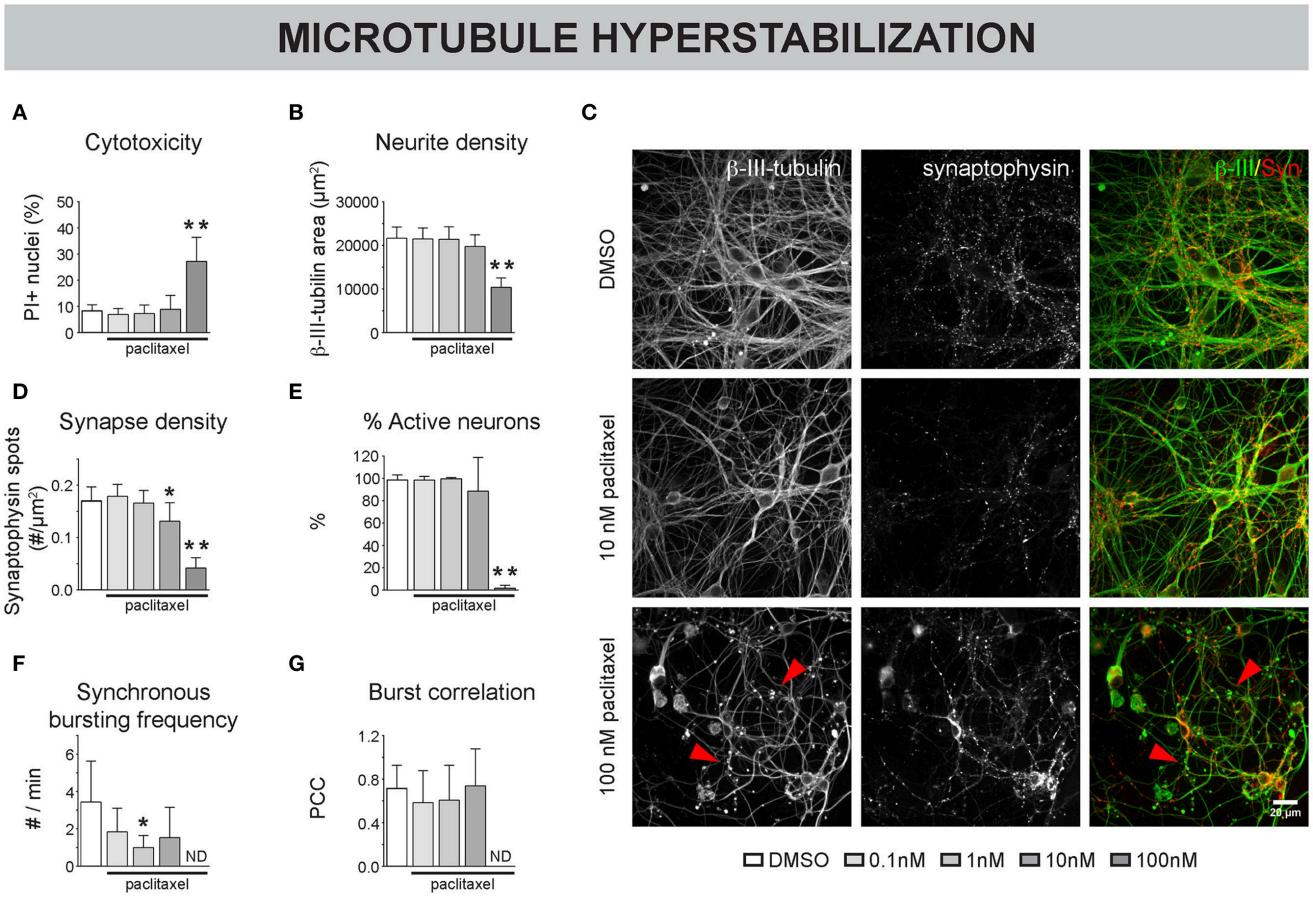
Pharmacological stabilization of microtubules (MTs) impairs neuronal network connectivity. **(A)** The percentage of propidium iodide (PI) -positive nuclei was quantified to measure cytotoxicity. Paclitaxel (4–7 DIV) treatment induced significant toxicity at a concentration of 100 nM (7 DIV; n_b_ = 3, n_w_ = 2). **(B)** This toxicity was accompanied by a reduction in neurite density, measured as the area of the neurite marker β-III-tubulin **(C)** Immunostaining of 7 DIV cultures for β-III-tubulin and the synapse marker synaptophysin revealed overt neurite pathology and debris (arrowheads) at 100 nM paclitaxel. **(D)** Quantification of synapse density showed a significant reduction by 10 and 100 nM paclitaxel. Exposure to 10 nM paclitaxel decreased synapse density while leaving the neurite network largely intact (n_b_ = 3, n_w_ = 2). **(E)** Live cell calcium imaging at 7 DIV showed a nearly complete loss of spontaneous activity at 100 nM paclitaxel. **(F,G)** Sub-toxic paclitaxel doses reduced the frequency of synchronized bursts, while the synchronicity of the remaining bursts across neurons (Burst correlation; PCC: Pearson's Correlation Coefficient) was not affected. The spontaneous activity was lost at 100 nM (ND: not determined since # active neurons/field of view <5; n_b_ = 4, n_w_ = 3). ^*^p < 0.05; ^**^p < 0.005.

To assess the impact of MT hyperstabilization on neuronal network connectivity, a dose-range study of paclitaxel (up to 100 nM) was performed on primary hippocampal cultures, treated from 4 to 7 DIV. A concentration of 100 nM proved to be cytotoxic as indicated by a significant increase in PI-positive cells (Figure 2A), overt neurite pathology (e.g., dilation, Figures 2B,C), and total absence of calcium oscillations (Figures 2E–G). A lower, non-toxic concentration of 10 nM paclitaxel significantly reduced synapse density (as measured by total number of synaptophysin-positive spots per μm^2^ neurite, Figure 2D), while leaving neurite density (as measured by total ß–tubulin area per field of view, Figure 2B) largely unchanged. A reduced frequency of synchronized bursts was observed for all subtoxic concentrations, but the reduction was only statistically significant for 1 nM paclitaxel (Figure 2F). Burst correlation, i.e., synchronicity of bursts across neurons, was not significantly affected (Figure 2G). Thus, the spontaneous network activity remained synchronous, albeit with longer burst intervals.

**Figure 3:**
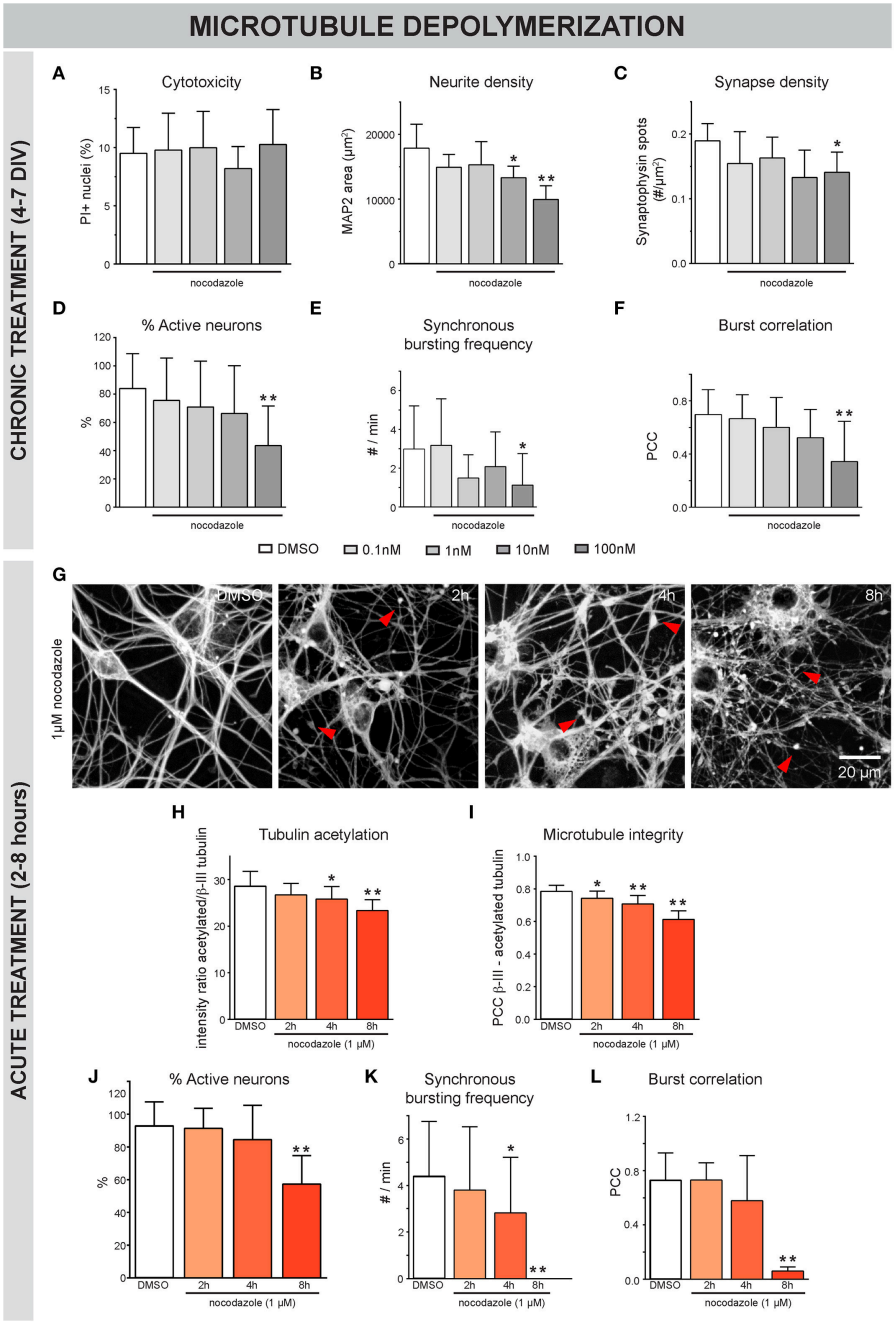
Nocodazole-induced MT depolymerization adversely affects morphofunctional neuronal connectivity. **(A)** Chronic (4–7 DIV) treatment with the MT-depolymerizing drug nocodazole did not induce significant toxicity, measured as the percentage of PI-positive nuclei, up to a concentration of 100 nM (n_b_ = 2, n_w_ = 4). **(B,C)** Quantification of neurite- and synapse density revealed a reduction in both parameters after exposure to 100 nM nocodazole. At a concentration of 10 nM, the neurite density was reduced while the synapse density was largely unaffected (n_b_ = 2, n_w_ = 4). **(D–F)** Functional connectivity was studied by means of live cell calcium imaging (n_b_ = 2, n_w_ = 4). The percentage of active neurons, frequency of synchronous bursts and burst correlation gradually decreased until statistically significant at a concentration of 100 nM nocodazole. **(G)** β-III-tubulin staining after acute nocodazole treatment (7 DIV, 2/4/8 h treatment), revealed progressive thinning of neurites and the appearance of tubulin patches (arrowheads). **(H)** Measurement of the intensity ratio of acetylated α-/β-III-tubulin showed a time-dependent reduction in tubulin acetylation, indicative of MT destabilization (n_b_ = 2, n_w_ = 4). **(I)** Colocalization of β-III- and acetylated α-tubulin was measured to quantify MT integrity with high sensitivity and showed progressive MT depolymerization (n_b_ = 2, n_w_ = 4). **(J)** Calcium imaging revealed a significant reduction in the percentage of active neurons, 8 h after nocodazole addition. **(K,L)** Synchronized activity was impaired in a time-dependent manner, with almost complete loss of synchrony after 8 h (n_b_ = 3, n_w_ = 4). ^*^p < 0.05; ^**^p < 0.005.

To assess the impact of MT destabilization, we next examined the effects of nocodazole (Figure 3). Chronic (4-7 DIV) treatment did not induce significant cytotoxicity up to a concentration of 100 nM (Figure 3A). However, neurite- and synapse density (Figures 3B,C), as well as the percentage of active neurons (Figure 3D) and synchronous bursting behavior (Figures 3E,F), were significantly impaired by 100 nM and, to a lesser extent, by 10 nM nocodazole. Acute addition of 1 μM nocodazole at 7 DIV resulted in a progressive thinning of neurites and the appearance of tubulin patches (Figure 3G, arrowheads) and was accompanied by reduced acetylation of α-tubulin (Figure 3H). Since stable, acetylated MTs are less sensitive to depolymerization, the correlation of β-III- and acetylated α-tubulin was used as a measure for MT integrity. This readout revealed a time-dependent decrease in MT integrity (Figure 3I). At the functional level, nocodazole destabilization induced a progressive loss of active neurons (Figure 3J) and synchronous calcium bursting behavior (Figures 3K,L).

These results indicate that hyperstabilization as well as depolymerization of neuronal MTs impair morphofunctional connectivity in primary culture. Therefore, we conclude that tight regulation of MT stability is important for maintaining synaptic connectivity in neuronal networks.

### Nocodazole-induced connectivity defects can be rescued by MT stabilizers

**Figure 4:**
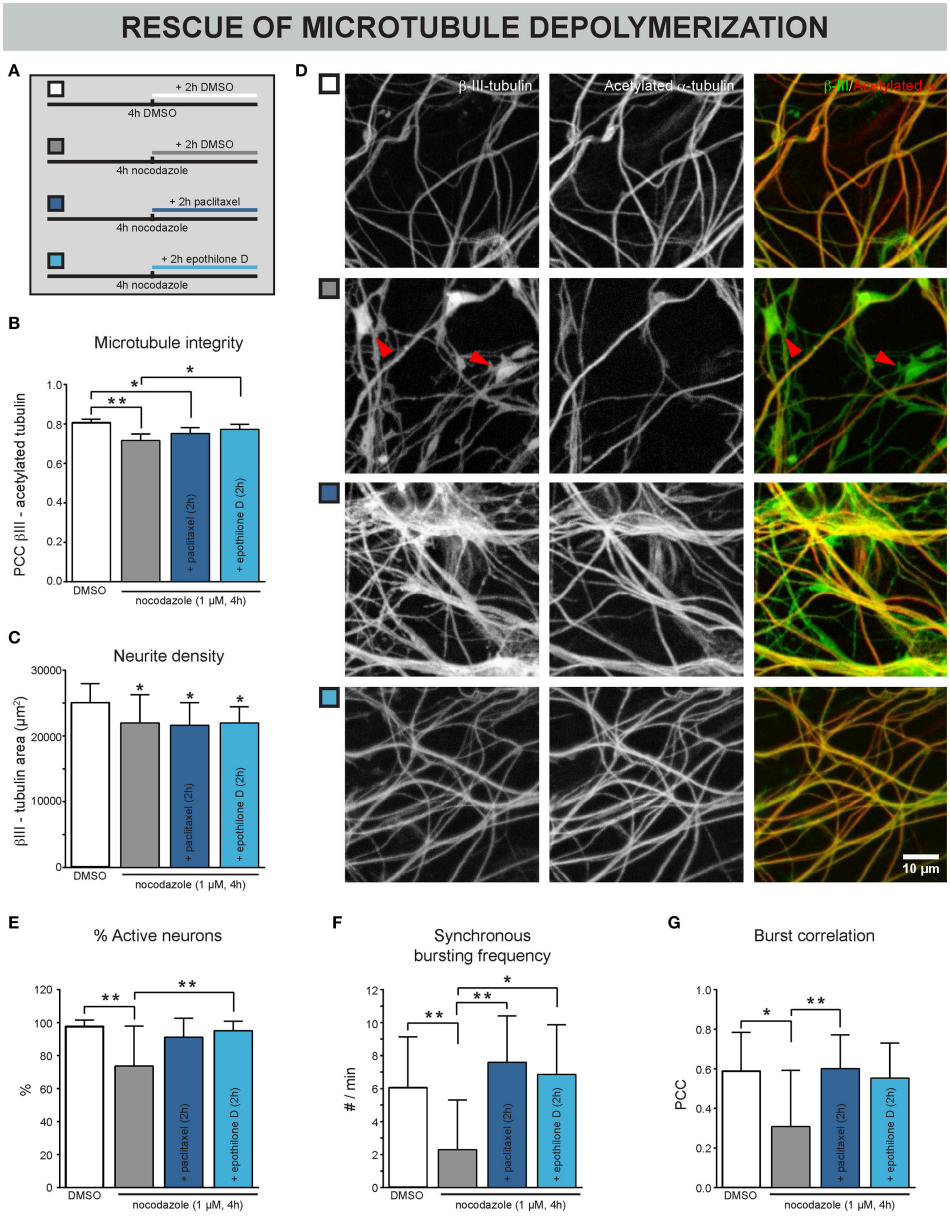
Nocodazole-induced impairment of neuronal network connectivity can be rescued by MT stabilizers. **(A)** Treatment scheme. The cultures were exposed to nocodazole for 4 h. During the last 2 of these 4 h, the MT stabilizers paclitaxel or epothilone D were added. DMSO was added as control. **(B)** A decreased correlation between β-III- and acetylated α-tubulin, a marker for the more stable population of MTs, was detected after 4 h nocodazole treatment, consistent with the staining in **(D)**. This phenotype could be partially rescued by adding paclitaxel (100 nM) and epothilone D (100 nM) during the last 2 h of nocodazole treatment (n_b_ = 3, n_w_ = 4). **(C)** Measurement of the β-III-tubulin area did not reveal any rescue of the nocodazole-induced reduction (n_b_ = 3, n_w_ = 4). **(D)** Immunostaining for β-III- and acetylated α-tubulin showed that nocodazole treatment (4 h) induced the formation of β-III-positive/acetylated α-negative tubulin patches (arrowheads; 7 DIV). This effect was rescued by adding the MT-stabilizing drugs paclitaxel or epothilone D during the last 2 h of nocodazole treatment. **(E)** A decreased percentage of neurons showed calcium activity after nocodazole treatment. This impairment could be rescued by subsequent MT re-stabilization (n_b_ = 3, n_w_ = 4). **(F,G)** While nocodazole treatment alone impaired the synchronous network activity, combination treatment with MT stabilizers showed similar functional connectivity to DMSO-treated controls (n_b_ = 3, n_w_ = 4). ^*^p < 0.05; ^**^p < 0.005.

Next, we sought to investigate whether chemically induced MT depolymerization could be rescued with MT stabilizers. Neurons were exposed to nocodzaole for 4 h. During the last 2 h of these 4 h, paclitaxel was added (Figure 4A). Epothilone D, a MT stabilizer with similar action but better BBB penetration than taxanes (Brunden et al., 2011), was included here to broaden the potential translational value of these experiments. DMSO was used as a control, both upon nocodazole and MT stabilizer treatment. As shown in previous experiments, nocodazole treatment for 4 h significantly reduced the Pearson's correlation coefficient (PCC, Figures 4B,D), pointing to a morphological breakdown of MTs. Conversely, addition of MT stabilizers 2 h after nocodazole incubation, (partly) rescued the nocodazole-induced PCC decrease (Figures 4B,D), while a decrease in neurite density, measured as the β-III-tubulin area, remained evident (Figure 4C). This combined treatment correlated with an improvement in functional connectivity, as evidenced by an increase in the percentage of active neurons (Figure 4E), synchronous bursting frequency (Figure 4F) and burst correlation (Figure 4G), in comparison with the nocodazole treatment alone.

These results show that chemically induced defects in MT-stability are reversible, at least in short-term experiments.

### MAPT overexpression induces connectivity defects that correlate with MT binding affinity

**Figure 5:**
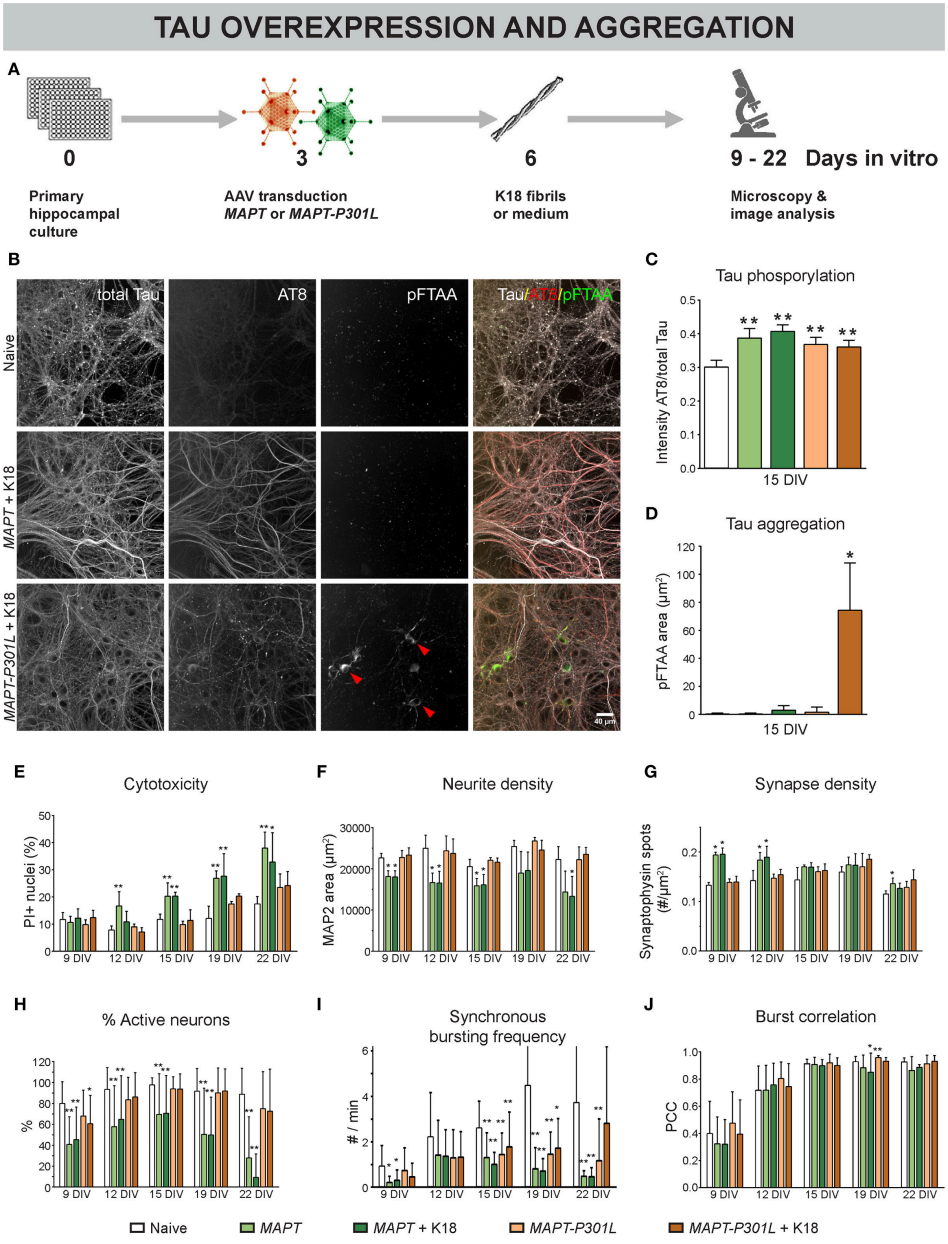
Overexpression of *MAPT* and *MAPT-P301L* induces phenotypically distinct defects in neuronal network connectivity. **(A)** Schematic representation of the treatment protocol. At 6 DIV, pre-formed Tau fibrils (K18) were added to half of the cultures as seeds for Tau aggregation. **(B)** Fluorescent labeling of 15 DIV cultures with an AT8 antibody for hyperphosphorylated Tau and pFTAA for fibrillar Tau. **(C,D)** *MAPT* and *MAPT-P301L* overexpression induced Tau hyperphosphorylation, but intracellular Tau aggregates were only detected upon *MAPT-P301L* overexpression and seeding with K18 fibrils (n_b_ = 2; n_w_ = 3). **(E)** Cytotoxicity, measured as the percentage of PI-positive nuclei, was detected at later time points and was more pronounced for *MAPT* than for *MAPT-P301L* overexpression, while K18 seeding did not exacerbate cell death (n_b_ = 2, n_w_ = 3). **(F,G)** Quantification of neurite- and synapse density after immunostaining for MAP2 and synaptophysin. While neurite density was decreased upon *MAPT* overexpression on all time points, a—plausibly compensatory—increase in synapse density was only seen at 9 and 12 DIV (n_b_ = 2, n_w_ = 3). **(H)** Live cell calcium imaging showed a reduction in the percentage of active neurons that was more pronounced for *MAPT* than for *MAPT-P301L* overexpression (n_b_ = 2, n_w_ = 6). **(I)** The frequency of synchronous bursts was decreased upon *MAPT* and *MAPT-P301L* overexpression. **(J)** The synchronicity of bursting was not consistently affected by any treatment. Addition of K18 fibrils did not alter the network activity. ^*^p < 0.05; ^**^p < 0.005.

To further study the role of MT dynamics in neuronal connectivity, we adopted a model for overexpression and intracellular aggregation of the MT-associated protein Tau in primary neurons (Figure 5A) (Guo and Lee, 2013). At 3 DIV, neurons were infected with AAV particles to trigger overexpression of human normal *MAPT* or mutant *MAPT-P301L*, resulting in overproduction of normal Tau or mutant Tau-P301L protein, respectively. The gene encoding the latter harbors a point mutation in the fourth MT-binding domain that decreases the affinity for MTs (Hong et al., 1998; Dayanandan et al., 1999). At 6 DIV part of these cultures were exposed to K18 fibrils to seed intracellular aggregation of Tau. Such fibrils consist of truncated P301L-4R-Tau, rich in β-sheets that induce aggregation (Siddiqua and Margittai, 2010). At 15 DIV, AT8 immunostaining showed higher Tau phosphorylation for both overexpression models, irrespective of K18 seeding, suggesting that the increased phosphorylation status is not sufficient to induce aggregation (Figure 5C). Intracellular Tau aggregates appeared as pFTAA-positive structures only in cells overexpressing *MAPT-P301L* and treated with K18 fibrils (Figures 5B,D; Supplemental Video 1). Tau aggregation was not observed in cultures that did not receive K18 fibrils or upon normal *MAPT* overexpression, indicating that the P301L gene product had an increased tendency to aggregate. At this MOI (100), overexpression of cytosolic eGFP (used as control) did not induce cytotoxicity, nor did it change the functional connectivity (Supplemental Figure 1). However, cytotoxicity measurements at different DIVs revealed the toxic effect of *MAPT* overexpression at later time points. This was not observed for *MAPT-P301L* overexpression (Figure 5E). K18 seeding did not exacerbate cytotoxicity in either model. Upon *MAPT* overexpression, a reduction in neurite density was observed for all time points (Figure 5F). A plausibly compensatory increase in synapse density occurred only at 9 and 12 DIV (Figure 5G). Functionally, *MAPT* overexpression impaired neuronal network activity, as confirmed by a decreased percentage of active neurons (Figure 5H) and a reduction in the frequency of synchronous bursts (Figure 5I). Corresponding to the cytotoxicity and morphology measurements, *MAPT* overexpression had a more profound effect on the percentage of active neurons than *MAPT-P301L* overexpression, and seeding with K18 fibrils did not exacerbate the connectivity impairment. In line with the observations that were made for paclitaxel, none of the treatments consistently altered the burst correlation (Figure 5J). As such, the activity remained synchronous, albeit depressed in frequency. Remarkably, the presence of Tau aggregates did not affect the neuron's ability to burst in synchrony with the surrounding network of unaffected neurons (Supplemental Figure 2).

These results show that *MAPT* overexpression induced more severe connectivity defects than *MAPT-P301L*, which corresponds with their respective binding affinity to MTs. They also demonstrate that K18 seeding does not exacerbate connectivity impairment, not even when intracellular Tau aggregation is induced.

### MT dynamics are differentially altered by Tau and Tau-P301L

**Figure 6:**
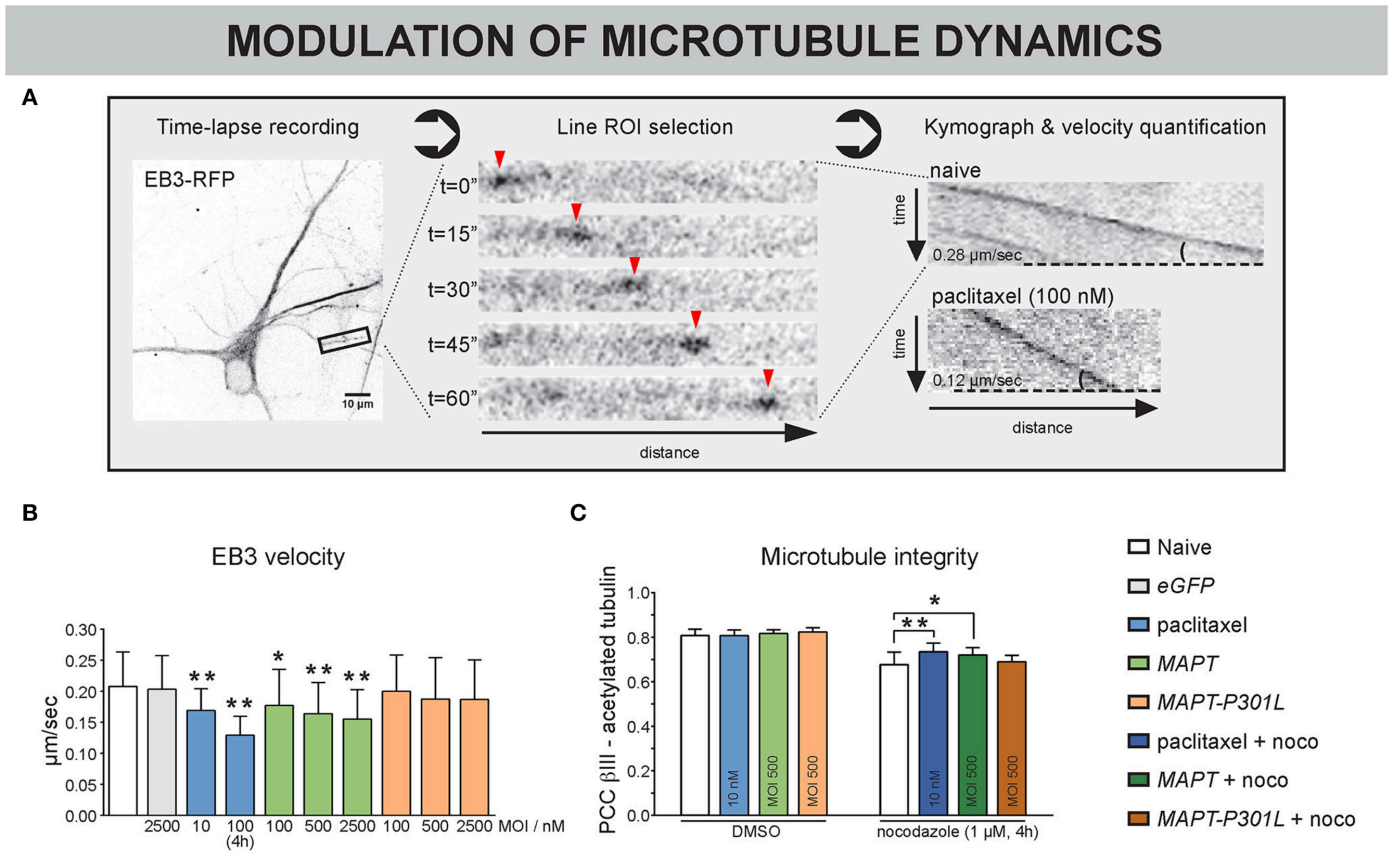
MT dynamics are differentially altered by *MAPT* and *MAPT-P301L* overexpression. **(A)** One-minute recordings (0.5 frames/min) of EB3-RFP overexpressing neurons were made to directly visualize the dynamics of MTs at 10 DIV. After selection of a stretch of at least 20 μm, a moving EB3 comet (arrowheads) can be discerned. Kymographs of an untreated vs. a paclitaxel treated neuron clearly show the difference in velocity, measured as the slope of the EB3 track. **(B)** Quantification showed reduced EB3 velocity in paclitaxel-treated neurons, which was less pronounced for long-term (10 nM, 3–10 DIV) than for short-term (100 nM, 4 h) treatment. Neurons overexpressing *MAPT* displayed a similar and dose-dependent reduction, which was absent upon *MAPT-P301L* overexpression (3–10 DIV, n_b_ = 2, n_w_ = 3, ≥ 7 segments/well). **(C)** MT integrity was quantified by measuring the colocalization of β-III- and acetylated α-tubulin. Though no difference in MT integrity was seen under basal conditions, paclitaxel treatment and *MAPT* overexpression protected against nocodazole-induced MT depolymerization, while *MAPT-P301L* overexpression did not (3–10 DIV; n_b_ = 2, n_w_ = 5). ^*^p < 0.05; ^**^p < 0.005.

To directly investigate MT dynamics in detail, the velocity of EB3-RFP comets, located at the MT plus ends, was quantified at 10 DIV (Figure 6A). To keep consistency with previous results and work in optimally interconnected networks, a low-efficiency transfection protocol was used to sparsely label isolated neurons within dense networks, rather than to e.g., isolate axons in microchannel-based cultivation systems (Kilinc et al., 2015). Paclitaxel treatment, which was used as a positive control, showed a decrease in EB3 velocity for chronic (10 nM, 3–10 DIV) and acute (100 nM, 4 h) exposure (Figures 6A,B). Similar to paclitaxel, *MAPT* overexpression dose-dependently decreased EB3 velocity; an effect that was not observed upon *MAPT-P301L* overexpression. Nocodazole treatment (1 μM, 4 h) caused all EB3 comets to vanish, plausibly because they disengaged from depolymerizing MT (Supplemental Video 2). Paclitaxel-treated neurons or neurons overexpressing *MAPT* or *MAPT-P301L* did not show any difference in correlation between β-III- and acetylated α-tubulin, suggesting no alteration in MT stability (Figure 6C). However, co-exposure of the neurons to nocodazole (1 μm, 4 h, 10 DIV), revealed significant differences, indicating differential sensitivity to depolymerization: overexpression of *MAPT*, much like pre-treatment with paclitaxel, partly protected against nocodazole-induced depolymerization, whereas *MAPT-P301L* overexpression did not.

Together, these data suggest that overexpression of *MAPT* induces a paclitaxel-like stabilization of neuronal MT and has an adjoined impact on neuronal connectivity (Figure 5), while *MAPT-P301L* overexpression does far less.

### Subtle MT depolymerization neither prevents nor rescues Tau-induced connectivity defects

**Figure 7:**
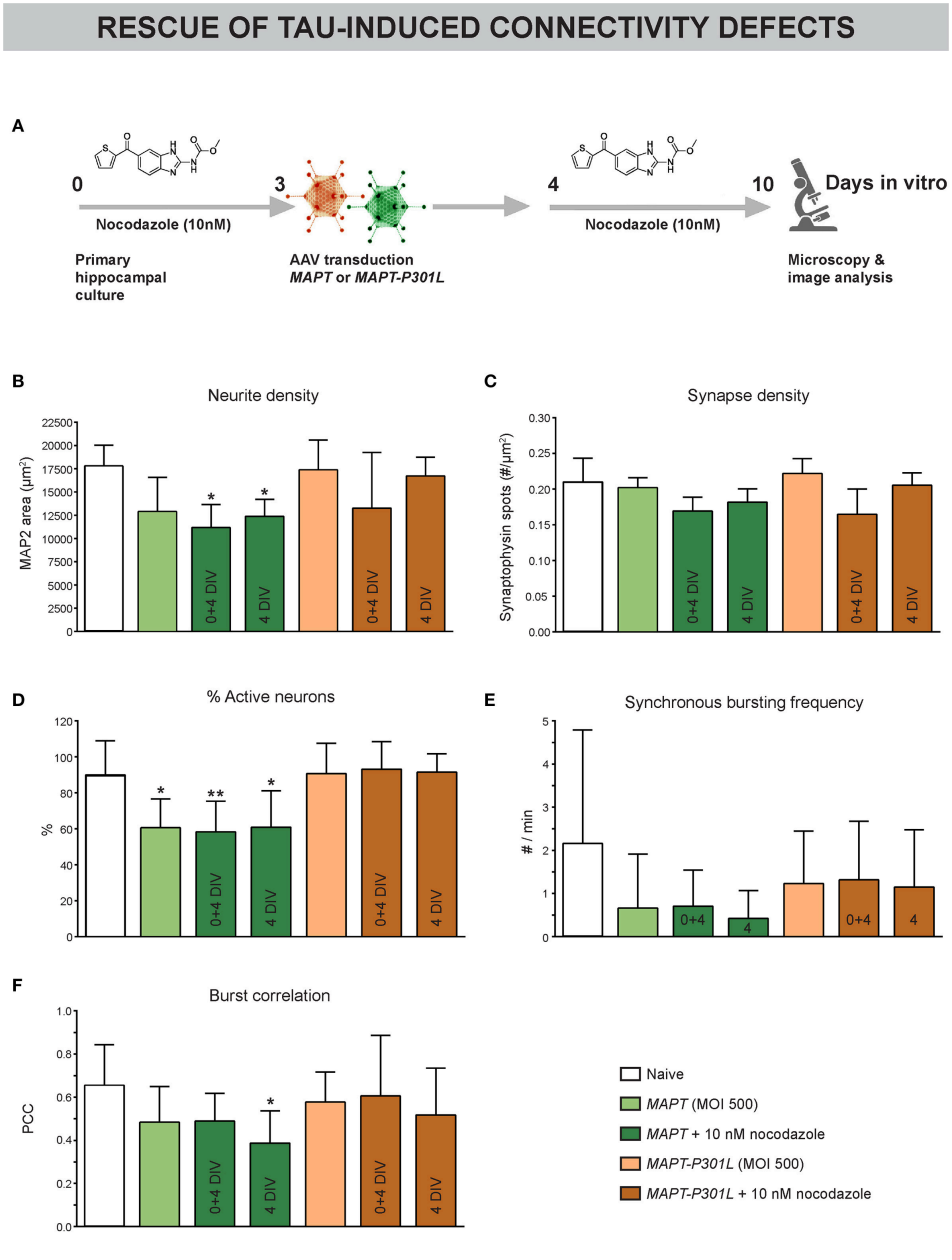
Subtle MT depolymerization neither prevents nor rescues Tau-induced connectivity defects. **(A)** Schematic representation of the treatment protocol. Nocodazole was added at 0+4 or 4 DIV. **(B)** Quantification of neurite outgrowth after MAP2 immunostaining showed a tendency toward reduced neurite density upon *MAPT* but not *MAPT-P301L* overexpression. Chronic nocodazole (pre-)treatment did not rescue but rather aggravated this defect (n_b_ = 2, n_w_ = 3). **(C)** Quantification of synaptophysin spots showed that synapse density was not affected by *MAPT* overexpression (n_b_ = 2, n_w_ = 3). **(D)** Live cell calcium imaging at 10 DIV showed a reduction in the percentage of active neurons upon *MAPT* overexpression that was not rescued by nocodazole (pre-)treatment (n_b_ = 2, n_w_ = 3). **(E,F)** A slight, but non-significant, reduction in the synchronous bursting frequency and burst correlation was detected upon *MAPT* overexpression. This reduction was less pronounced in the case of *MAPT-P301L* overexpression and could not be rescued by chronic nocodazole treatment (n_b_ = 2, n_w_ = 3). ^*^p < 0.05; ^**^p < 0.005.

Since *MAPT* overexpression was found to induce paclitaxel-like stabilization of neuronal MTs, we tested the potential of long-term nocodazole treatment to prevent (treatment at 0 + 4 DIV) or rescue (from 4 DIV onwards) the associated connectivity defects (Figure 7A). Morphological interrogation showed a tendency toward decreased neurite density in neurons overexpressing *MAPT* but not *MAPT-P301L* (Figure 7B). Long-term preventive or rescue treatment with a low nocodazole concentration did not alleviate this phenotype, but rather aggravated it. Synapse density was not affected in any condition (Figure 7C). Calcium imaging showed a reduction in the percentage of active neurons for *MAPT* overexpression only. But, this defect was not improved by nocodazole (pre-)treatment (Figure 7D). Corresponding to this observation, a tendency toward decreased synchronized activity upon *MAPT* overexpression was seen that was less pronounced for *MAPT-P301L* overexpression (Figures 7E,F). Also in these parameters, no beneficial effect of nocodazole was detected.

These data show that Tau-induced connectivity defects cannot be rescued by nocodazole treatment.

## Discussion

Neuronal MTs support many functions that are important for synaptic connectivity, such as neurite outgrowth, polarized cargo transport and local signaling at the synapse (Gardiner et al., 2011; Matamoros and Baas, 2016). Using functionally connected primary neurons, we showed that positive (paclitaxel) as well as negative (nocodazole) pharmacological modulation of MT stability impairs morphofunctional connectivity. It was shown before that paclitaxel inhibits both the shortening and growth rates, and hence the dynamic instability of MTs in cancer cells (Yvon et al., 1999). We now confirmed a decreased velocity of EB3-RFP comets and an enhanced protection against depolymerization in primary hippocampal neurons. Paclitaxel-treated cultures also showed impaired morphofunctional connectivity, which was already apparent by calcium imaging, at concentrations as low as 1 nM. In addition to MT hyperstabilization by paclitaxel, long- and short-term nocodazole treatment also impaired morphofunctional connectivity in these primary hippocampal neurons. Short-term treatment induced the disappearance of EB3-RFP comets, thinning of neurites and the appearance of tubulin patches in the vicinity of neurites, recapitulating the hallmarks of MT depolymerization. Perturbation of synchronous calcium bursting by nocodazole was detected after 4 h, while immunostaining for β-III-tubulin showed breakdown of MTs already at earlier time points. Although more research is needed to uncover the exact reason for this time lag, one could hypothesize that the synapse is self-sufficient for a limited amount of time as a result of synaptic vesicle recycling. In support of this hypothesis, super-resolution microscopy experiments revealed that nocodazole affects the transport of synaptic vesicles exclusively in neurites but not in synapses (Maschi and Klyachko, 2015). From this set of pharmacology experiments, we concluded that strict regulation of MT dynamics is essential for maintaining synaptic connectivity in primary hippocampal cultures.

We showed that paclitaxel pre-treatment reduced morphological MT breakdown, and subsequent treatment rescued nocodazole-induced connectivity defects. Furthermore, we successfully included epothilone D in the nocodazole rescue experiments. This compound stabilizes MT in a paclitaxel-like fashion (Alberti, 2013), but has better blood-brain-barrier penetration, making it a more attractive therapeutic compound for the CNS (Brunden et al., 2011). Since we expect the differences between paclitaxel and epothilone D to only become evident in *in vivo* experiments, we did not fully characterize the effect of epothilone D on morphofunctional connectivity *in vitro*. Nevertheless, the fact that nocodazole-induced connectivity defects could be rescued by epothilone D broadens the potential translational value of our findings. It remains, however, difficult to conclude whether MTs represent druggable targets in disorders characterized by impaired neuronal connectivity, since we also showed that MT hyperstabilization (Figure 2) and depolymerization (Figure 3) compromised morphofunctional connectivity. As such, it might prove difficult to determine a safe dose and timing scheme for such treatment.

To further confirm the hypothesis that MT dynamics determine the level of synaptic connectivity, we switched to a disease model relevant for tauopathies. In physiological conditions, the MT-associated protein Tau stabilizes MTs by binding to the interface between tubulin heterodimers (Kadavath et al., 2015). We found that overexpression of *MAPT* in hippocampal neurons led to cytotoxicity and impaired network activity. Given the observation that *MAPT* overexpressing neurons, just like paclitaxel-treated neurons, were more resistant to nocodazole-induced depolymerization and displayed lower EB3 velocity, we conclude that the negative impact on synaptic connectivity is at least partly the result of MT hyperstabilization. This was further confirmed by the observation that *MAPT-P301L* overexpression evoked less pronounced connectivity defects. Several groups previously reported that Tau-P301L displayed reduced affinity for MTs in biochemical assays (Hong et al., 1998; Barghorn et al., 2000; Fischer et al., 2007) or in cell lines (Dayanandan et al., 1999; DeTure et al., 2000; Lu et al., 2000). Although the above-mentioned observations strongly suggest a link between Tau-induced MT hyperstabilization and connectivity impairment, mild nocodazole depolymerization did not prevent nor rescue such defects. Although we cannot exclude that other treatment schemes might reveal such rescue, the current data suggest that MT stability is not the sole target of *MAPT* overexpression. In line with this, next to its MT-binding capacity, Tau has been shown to modulate chromatin relaxation, translation initiation and mitochondrial function (Eckert et al., 2014; Frost et al., 2014; Meier et al., 2016). Nevertheless, a broader range of nocodazole concentrations and treatment times should be tested to fully support this conclusion.

The amino acid residues responsible for MT binding are also essential for the pathological aggregation of Tau (Mukrasch et al., 2005; Kadavath et al., 2015). We adopted an *in vitro* model based on the induction of aggregation by preformed K18 Tau fibrils (Guo and Lee, 2013). Consistent with literature, we found that only *MAPT-P301L* but not *MAPT* overexpressing neurons showed formation of intracellular Tau aggregates after K18 seeding (Guo and Lee, 2013). A combination of conformational changes and reduced MT binding are believed to underlie this property (Barghorn et al., 2000; von Bergen et al., 2001; Terwel et al., 2005). Surprisingly, segregated analysis of calcium signals in *MAPT-P301L* overexpressing neurons with and without intracellular Tau aggregates did not expose an aggravated phenotype in the former. One explanation for this could be that synaptotoxicity results from oligomeric hyperphosphorylated Tau, rather than from insoluble tangles (Hoover et al., 2010; Cowan and Mudher, 2013; Guerrero-Munoz et al., 2015; Kruger and Mandelkow, 2016). The sudden exposure to pre-formed Tau fibrils may bypass the slow oligomerization phase that normally precedes Tau aggregation (Guo and Lee, 2014). Alternatively, the background expression levels of normal *MAPT* may mask *MAPT-P301L*-dependent effects. The recent advent of CRISPR/Cas9 genome editing technology (Ran et al., 2013) may lead to the generation of more accurate mouse models. Such models might disclose subtle phenotypic alterations that are more difficult to detect in the overexpression model that was used in this study.

Like every *in vitro* model, the neuronal networks that were used in this study have obvious limitations. The cells are grown in 2D, they lack the different cell types and specific wiring pattern as seen in the hippocampus, and they are devoid of external input. The results obtained with this system should therefore be validated in animal models and eventually in men. From a translational perspective, chronic administration of low doses of epothilone D was found to improve neuropathology and cognitive performance in several tauopathy mice (Brunden et al., 2010; Barten et al., 2012; Zhang et al., 2012). Similar results were obtained in *C. elegans* and *Aplysia* (Shemesh and Spira, 2011; Erez et al., 2014; Miyasaka et al., 2016). Nevertheless, one phase I clinical trial to assess the safety, tolerability and effect of low doses of epothilone D in subjects with mild AD did not proceed to phase II (http://www.clinicaltrials.gov/ct/show/NCT01492374). The results of this specific trial remained unpublished, but in general, gaps in the fundamental knowledge are causing the high failure rates in AD-related clinical trials. In light of this, the current study contributes to the basic understanding of the role of MT stability in neuronal connectivity. We used primary hippocampal neurons that, in contrast to immortal neuronal cell lines such as neuroblastoma cells, retain several *in vivo* properties like spontaneous neurite outgrowth, synapse formation and, most importantly, synchronized electrical activity. The translational value of this model may even be further improved by switching to a fully humanized system. It was recently shown that human iPSC-derived neurons develop synchronized network activity and can be interrogated for morphofunctional connectivity (Kuijlaars et al., 2016). Though current reprogramming protocols are tedious and time-consuming as compared to cultivating mouse primary neurons, they can even be derived from patients with sporadic forms of the disease (Armijo et al., 2017) and can be combined with gene editing approaches (Paquet et al., 2016).

In conclusion, we have used an *in vitro* approach to expose the relevance of MT stability in maintaining neuronal network connectivity. Despite proof for reversible pharmacological tuning, a subtle balance and dose and time dependency currently hamper fast translational progress for this potential therapeutic target.

## Author contributions

PV, JD, JK, RN, JT, and WD conceived and designed the experiments. PV and JD performed the experiments. PV, JD, MV, and WD analyzed the data. PV and WD drafted the manuscript. All authors critically revised the manuscript and are accountable for all aspects of the work.

### Conflict of interest statement

The authors declare that the research was conducted in the absence of any commercial or financial relationships that could be construed as a potential conflict of interest.

## Abbreviations

MT: microtubule
PI: propidium iodide
PCC: Pearson's Correlation Coefficient.
