# Supplementary material for: Dysregulation of Microtubule Stability Impairs Morphofunctional Connectivity in Primary Neuronal Networks
Source: Front Cell Neurosci. 2017 Jun 22;11:173. doi: 10.3389/fncel.2017.00173 (PMC5480095; doi:10.3389/fncel.2017.00173)
Supplement: Supplementary file 1 [file Table1.PDF]

Fig	description	DIV	# observations/well	n_{well}	$n_{biological}$	# data points/ treatment group	Shapiro-Wilk	Kruskal-Wallis	post-hoc test
2A	paclitaxel: cytotoxicity	7	16 images/well	2	3	6	p<0.0001	p=0.0017	Steel
2B	paclitaxel: neurite density	7	16 images/well	2	3	6	p<0.0001	p<0.0001	Steel
2D	paclitaxel: synapse density	7	16 images/well	2	3	6	p<0.0001	p<0.0001	Steel
2E	paclitaxel: % active neurons	7	1 calcium recording/well	3	4	12	p<0.0001	p<0.0001	Steel
2F	paclitaxel: synchronous bursting frequency	7	1 calcium recording/well	3	4	12	p=0.0015	p=0.0125	Steel
2G	paclitaxel: correlation	7	1 calcium recording/well	3	4	12	p=0.0009	p=0.4818	/
3A	nocodazole chronic: cytotoxicity	7	16 images/well	4	2	8	p=0.0093	p=0.5771	/
3B	nocodazole chronic: neurite density	7	16 images/well	4	2	8	p=0.8886	p=0.0005	Steel
3C	nocodazole chronic: synapse density	7	16 images/well	4	2	8	p=0.0792	p=0.0402	Steel
3D	nocodazole chronic: % active neurons	7	1 calcium recording/well	4	2	8	p<0.0001	p=0.0068	Steel
3E	nocodazole chronic: synchronous bursting frequency	7	1 calcium recording/well	4	2	8	p<0.0001	p=0.0342	Steel
3F	nocodazole chronic: correlation	7	1 calcium recording/well	4	2	8	p=0.0003	p=0.003	Steel
3H	nocodazole acute: acetylation	7	16 images/well	4	2	8	p=0.3031	p<0.0001	Steel
3I	nocodazole acute: MT integrity	7	16 images/well	4	2	8	p=0.0074	p<0.0001	Steel
3J	nocodazole acute: % active neurons	7	1 calcium recording/well	4	3	12	p<0.0001	p=0.0002	Steel
3K	nocodazole acute: synchronous bursting frequency	7	1 calcium recording/well	4	3	12	p=0.0009	p<0.0001	Steel
3L	nocodazole acute: correlation	7	1 calcium recording/well	4	3	12	p<0.0001	p=0.0003	Steel
4B	nocodazole rescue: MT integrity	7	16 images/well	4	3	12	p=0.0044	p<0.0001	Dunn
4C	nocodazole rescue: neurite density	7	16 images/well	4	3	12	p=0.0570	p=0.0031	Dunn
4E	nocodazole rescue: % active neurons	7	1 calcium recording/well	4	3	12	p<0.0001	p<0.0001	Dunn
4F	nocodazole rescue: synchronous bursting frequency	7	1 calcium recording/well	4	3	12	p=0.0006	p<0.0001	Dunn
4G	nocodazole rescue: correlation	7	1 calcium recording/well	4	3	12	p<0.0001	p=0.0021	Dunn
5C	Tau: phosphorylation	15	16 images/well	3	2	6	p=0.1932	p<0.0001	Steel
5D	Tau: aggregation	15	16 images/well	3	2	6	p<0.0001	p=0.0006	Steel
5E	Tau: cytotoxicity	9	16 images/well	3	2	6	p=0.2090	p=0.5434	/
		12	16 images/well	3	2	6	p=0.002	p=0.0322	Steel
		15	16 images/well	3	2	6	p=0.2078	p=0.0086	Steel
		19	16 images/well	3	2	6	p=0.4726	p=0.0027	Steel
		22	16 images/well	3	2	6	p=0.0578	p=0.0155	Steel
5F	Tau: neurite density	9	16 images/well	3	2	6	p=0.2325	p=0.0003	Steel
		12	16 images/well	3	2	6	p=0.0449	p=0.0004	Steel
		15	16 images/well	3	2	6	p=0.0099	p=0.0003	Steel
		19	16 images/well	3	2	6	p=0.0002	p=0.0029	Steel
		22	16 images/well	3	2	6	p=0.0021	p=0.0012	Steel
5G	Tau: synapse density	9	16 images/well	3	2	6	p=0.0007	p=0.0003	Steel
		12	16 images/well	3	2	6	p=0.3131	p=0.0003	Steel
		15	16 images/well	3	2	6	p=0.0002	p=0.1186	/
		19	16 images/well	3	2	6	p=0.9616	p=0.1137	/
		22	16 images/well	3	2	6	p=0.3581	p=0.0207	Steel
5H	Tau: % active	9	2 calcium recordings/well	6	2	12	p<0.0001	p<0.0001	Steel
		12	2 calcium recordings/well	6	2	12	p<0.0001	p=0.0002	Steel
		15	2 calcium recordings/well	6	2	12	p<0.0001	p=0.0009	Steel
		19	2 calcium recordings/well	6	2	12	p<0.0001	p<0.0001	Steel
		22	2 calcium recordings/well	6	2	12	p<0.0001	p<0.0001	Steel
5I	Tau: synchronous bursting frequency	9	2 calcium recordings/well	6	2	12	p<0.0001	p=0.0054	Steel
		12	2 calcium recordings/well	6	2	12	p<0.0001	p=0.1352	/
		15	2 calcium recordings/well	6	2	12	p<0.0001	p<0.0001	Steel
		19	2 calcium recordings/well	6	2	12	p<0.0001	p<0.0001	Steel
		22	2 calcium recordings/well	6	2	12	p<0.0001	p<0.0001	Steel
5J	Tau: correlation	9	2 calcium recordings/well	6	2	12	p=0.0002	p=0.1863	/
		12	2 calcium recordings/well	6	2	12	p<0.0001	p=0.2013	/
		15	2 calcium recordings/well	6	2	12	p<0.0001	p=0.0851	/
		19	2 calcium recordings/well	6	2	12	p<0.0001	p<0.0001	Steel
		22	2 calcium recordings/well	6	2	12	p<0.0001	p=0.1655	/
6B	EB3 velocity	10	≥7 segments/well	3	2	6	p<0.0001	p<0.0001	Steel
6C	MT integrity: DMSO	10	16 images/well	5	2	10	p=0.9851	p=0.2218	/
	MT integrity: nocodazole	10	16 images/well	5	2	10	p=0.0034	p=0.0016	Dunn
7B	Tau rescue: neurite density	10	16 images/well	3	2	6	p=0.0366	p=0.0004	Steel
7C	Tau rescue: synapse density	10	16 images/well	3	2	6	p=0.3751	p=0.0040	Steel
7D	Tau rescue: % active neurons	10	2 calcium recordings/well	3	2	6	p<0.0001	p<0.0001	Steel
7E	Tau rescue: synchronous bursting frequency	10	2 calcium recordings/well	3	2	6	p<0.0001	p=0.2168	/
7F	Tau rescue: Correlation	10	2 calcium recordings/well	3	2	6	p=0.7064	p=0.0119	Steel
S1A	Tau controls: cytotoxicity	9	16 images/well	3	2	6	p=0.5690	p=0.5754	/
		12	16 images/well	3	2	6	p=0.9983	p=0.3574	/
		15	16 images/well	3	2	6	p=0.8899	p=0.8134	/
		19	16 images/well	3	2	6	p=0.0155	p=0.8974	/
		22	16 images/well	3	2	6	p=0.4170	p=0.3542	/
S1B	Tau controls: % active neurons	9	2 calcium recordings/well	4	2	8	ND	p=1.0000	/
		12	2 calcium recordings/well	4	2	8	p<0.0001	p=0.3515	/
		15	2 calcium recordings/well	4	2	8	ND	p=1.0000	/
		19	2 calcium recordings/well	4	2	8	ND	p=1.0000	/
		22	2 calcium recordings/well	4	2	8	ND	p=1.0000	/
S1C	Tau controls: synchronous bursting frequency	9	2 calcium recordings/well	4	2	8	p<0.0001	p=0.9326	/
		12	2 calcium recordings/well	4	2	8	p<0.0001	p=0.4594	/
		15	2 calcium recordings/well	4	2	8	p<0.0001	p=0.8851	/
		19	2 calcium recordings/well	4	2	8	p=0.1034	p=0.5600	/
		22	2 calcium recordings/well	4	2	8	p=0.0005	p=0.3451	/
S1D	Tau controls: correlation	9	2 calcium recordings/well	4	2	8	p=0.0020	p=0.5450	/
		12	2 calcium recordings/well	4	2	8	p<0.0001	p=0.3181	/
		15	2 calcium recordings/well	4	2	8	p<0.0001	p=0.8947	/
		19	2 calcium recordings/well	4	2	8	p<0.0001	p=0.8696	/
		22	2 calcium recordings/well	4	2	8	p<0.0001	p=0.4119	/
