# Supplementary material for: Dysregulation of Microtubule Stability Impairs Morphofunctional Connectivity in Primary Neuronal Networks
Source: Front Cell Neurosci. 2017 Jun 22;11:173. doi: 10.3389/fncel.2017.00173 (PMC5480095; doi:10.3389/fncel.2017.00173)
Supplement: Supplementary file 4 [file DataSheet1.DOCX]

Supplementary Material

Dysregulation of microtubule stability impairs morphofunctional connectivity in primary neuronal networks.

Peter Verstraelen, Jan R. Detrez, Marlies Verschuuren, Jacobine Kuijlaars, Rony Nuydens, Jean-Pierre Timmermans and Winnok H. De Vos^*^

*** Correspondence:** Prof. Dr. Winnok De Vos: winnok.devos@uantwerpen.be

## Supplementary Figures


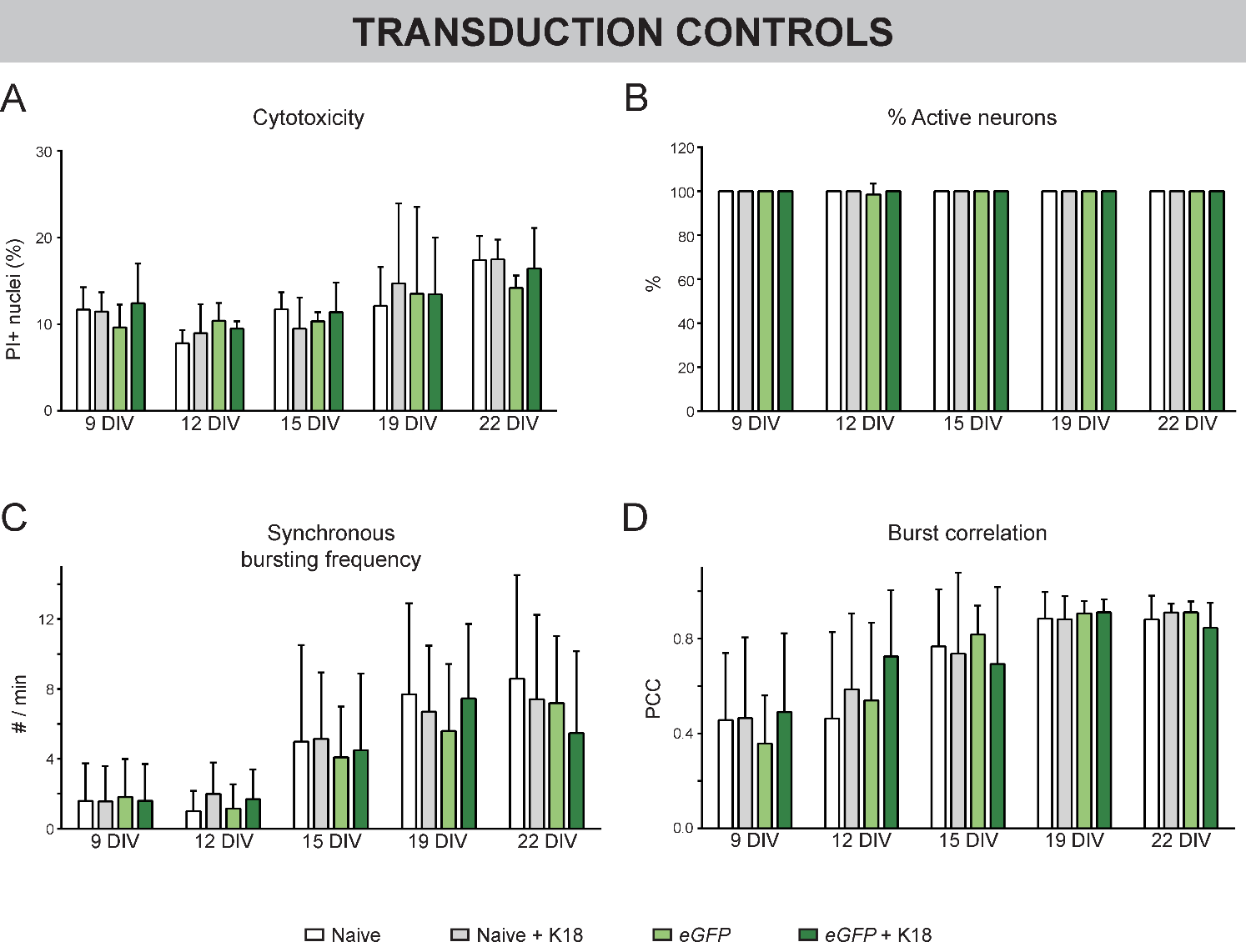


**Supplemental Figure 1.** **Cytosolic eGFP overexpression and K18 seeding do not alter cell viability or functional connectivity.** (A) Cyotoxicity was assessed by quantifying the percentage of propidium iodide (PI)-positive nuclei. Cytosolic eGFP overexpression (AAV transduction control) or K18 seeding did not increase toxicity (n_b_ = 2, n_w_ = 3). (B-D) Live cell calcium imaging was used as a sensitive readout for functional connectivity. The tested conditions did not alter the number of active neurons or the synchronous calcium bursting behavior (n_b_ = 2, n_w_ = 4).

**
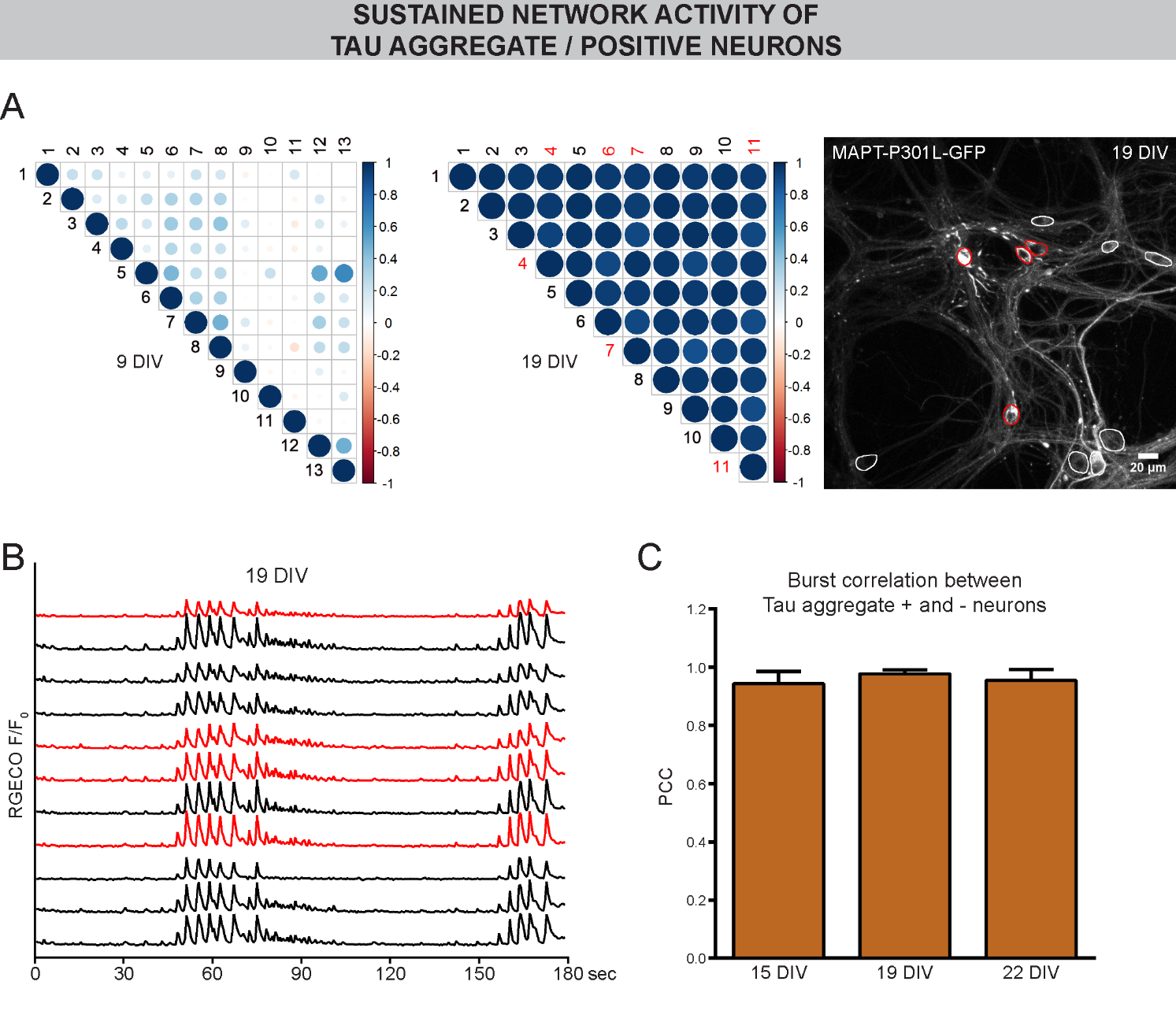
**

**Supplemental Figure 2: Neurons containing intracellular Tau aggregates do not show defects in synchronous bursting behavior.** (A) Correlograms of neurons that displayed asynchronous calcium bursting behavior at 9 DIV and synchronous activity at 19 DIV. The burst correlation between neuron pairs is depicted as the size and the color of the disks. Both were recorded from neurons with *MAPT-P301L* overexpression and K18 seeding. At 19 DIV, the numbers in red are neurons with somatic Tau aggregates, as also indicated on the *MAPT-P301L-eGFP* image. (B) RGECO traces of the 19 DIV recording show the high correlation between aggregate-positive (red) and aggregate-negative (black) neurons. (C) Quantification of the correlation between Tau aggregate-positive and –negative neurons within the same field-of-views showed a consistently high correlation between neurons with Tau inclusions and the surrounding network at different DIV (n_b_ = 2, n_w_ = 6).

## Supplementary Videos

**Supplemental Video 1: Timelapse recording of Tau aggregation in primary hippocampal neurons.** Neurons overexpressing *MAPT-P301L-eGFP* were exposed to K18 fibrils from 6 DIV to induce aggregation. The time-lapse recording was started at 16 DIV and lasted until 19 DIV. Four neurons showed intracellular accumulation of Tau aggregates. See also figure 5 for calcium imaging data.

**Supplemental Video 2: Timelapse recording of EB3-RFP in control and nocodazole-treated neurons.** While moving EB3-RFP comets can be discerned in the DMSO-treated neuron, the nocodazole-treated (1 µM, 4h) neuron shows diffuse cytoplasmic labeling instead of moving spots, plausibly because EB3 disengaged from depolymerizing MT.

## Supplementary Table

**Supplemental Table 1: Statistics** For each figure panel that contained quantitative data, the DIV, number of observations per well, the number of wells and the number of biological replicates is listed. When multiple observations were made within one well, the data were averaged per well before performing statistical analyses. One biological replicate refers to the dissection of one mother mice, of which the hippocampi from different embryos were pooled. The number of data points for each treatment group can be inferred by multiplying well and biological replicates. P-values of Shapiro-Wilk tests for normality are reported (ND: not determined since all values equaled 100). Since most of the data was not normally distributed (p<0.05), only non-parametric testing was done uniformly throughout the paper. Kruskal-Wallis (rank sums) tests were performed to assess the overall effect across treatments. Post-hoc testing was done using Steel tests for comparison with control, or Dunn all pairs tests for joint ranks. No post-hoc tests were carried out when the overall Kruskal-Wallis test returned non-significant p-values.
